# Progression of function and pain relief as indicators for returning to sports after arthroscopic isolated type II SLAP repair—a prospective study

**DOI:** 10.1186/s12891-017-1620-3

**Published:** 2017-06-13

**Authors:** Sandra Boesmueller, Thomas M. Tiefenboeck, Marcus Hofbauer, Adam Bukaty, Gerhard Oberleitner, Wolfgang Huf, Christian Fialka

**Affiliations:** 1 AUVA Trauma Center Vienna Meidling, Kundratstraße 37, 1120 Vienna, Austria; 2 Department of Trauma Surgery, Medical University of Vienna, Waehringer Guertel 18-20, 1090 Vienna, Austria; 3 Division of General Anaesthesia and Intensive Care Medicine, Medical University of Vienna, Waehringer Guertel 18-20, 1090 Vienna, Austria; 4 Department of Trauma Surgery, Wilhelminen Hospital Vienna, Montleartstraße 37, 1160 Vienna, Austria; 5 Center for Medical Physics and Biomedical Engineering, Medical University of Vienna, Waehringer Guertel 18-20, 1090 Vienna, Austria

**Keywords:** SLAP II, Repair, Functional outcome, Pain relief, Return to sports

## Abstract

**Background:**

One of the currently used surgical techniques in isolated type II SLAP lesions is arthroscopic SLAP repair. Postoperatively, patients tend to suffer from a prolonged period of pain and are restricted in their sports activities for at least 6 months. The aim of this study was to prospectively evaluate the clinical outcome as well as the postoperative course of pain after arthroscopic type II SLAP repair.

**Methods:**

Outcome measures were assessed using the Individual Relative Constant Score (CS_indiv_), the American Shoulder and Elbow Surgeons (ASES) Score, the Visual Analogue Scale (VAS), and the Short Form 36 (SF-36). Data were collected preoperatively, as well as at 3, 6, 12 and >24 months postoperatively.

**Results:**

Eleven patients with an average age of 31.8 years (range: 22.8-49.8 years) underwent arthroscopic repair of isolated type II SLAP lesions. Mean follow-up time was 41.9 months (range: 36.1–48.4 months). 6 months after surgery, there was a statistically significant improvement of function according to the CS_indiv_ (*p* = 0.004), the ASES Score (*p* = 0.006), and the SF-36 subscale “physical functioning” (*p* = 0.014) and a statistically significant decrease of pain according to the VAS (*p* = 0.007) and the SF-36 subscale “bodily pain” (*p* = 0.022) compared to preoperative levels.

**Conclusions:**

Arthroscopic repair of isolated type II SLAP lesions with suture anchors leads to a satisfactory functional outcome and return to pre-injury sports levels, with delayed, but significant pain relief observed 6 months after surgery. Thus, a return to sports should not be allowed earlier than 6 months after surgery, when patients have reached pain-free function and recovered strength.

**Trial registration:**

Researchregistry1761 (UIN).

## Background

Lesions to the long head of the biceps tendon were first described in baseball players by James Andrews in 1985 [1]. 5 years later, Steven Snyder coined the term “SLAP” (i.e., superior labrum anterior to posterior) lesions [2] – which have been shown to either occur from a fall on the flexed and abducted arm or from repetitive traction injuries in the overhead athlete.

According to the Snyder classification, 4 types of SLAP lesions are described – type II lesions being the most common, with an incidence of 55%. In an analysis of 140 injuries, Snyder observed that an isolated type II SLAP lesion was found in one third of patients [3], though most of the lesions (up to 88% of cases) were shown to have occurred with concomitant shoulder pathologies (e.g., Bankart lesions, rotator cuff tears or osteoarthritis of the humeral head) [3, 4].

In the literature, various techniques have been developed to stabilize type II lesions. Altchek et al. [5] reported good short-term functional results following debridement – however, the results deteriorated over time [5]. As such, in cases of type II SLAP lesions in young and active patients, fixation of the long head of the biceps tendon is recommended in order to restore the anatomical structures of the shoulder joint. There are, however, other techniques – such as biceps tenodesis or tenotomy [6, 7] – which yield good-to-excellent results, and with earlier pain relief.

Currently, only a few prospective studies are available which present data concerning functional outcome and return to sports following repair of isolated type II SLAP lesions at a final follow-up of at least 24 months [8–14]. The pain component itself is rarely addressed, or only compared to the outcome after biceps tenodesis or tenotomy [7, 15]. Although pain assessments of up to 50% of the scores are built into some instruments like the American Shoulder and Elbow Surgeons Standardized Shoulder Assessment Form (ASES) [16] or the Individual Relative Constant Score (CS_indiv_) [17] it is not possible to deduce the pain component from the total score, thus making a comparison of pain relief after surgery difficult.

Therefore, the purpose of this study was to evaluate the progression of function and pain relief after arthroscopic isolated type II SLAP repair in active patients. We hypothesized that there would be a significant improvement in function and reduction of pain over a postoperative observation period of two years, and that the ability to return to pre-injury sports activities is in the first line determined by regaining a pain-free condition, a full range of motion (ROM) and adequate strength. We furthermore hypothesized that the achievement of all these factors indicates the appropriate time point for a return to sports, which we expect to be - in most cases - not earlier than 6 months after surgery.

## Methods

### Patients

From June 2010 to June 2011, a total of 84 patients were treated at our outpatient clinic for an injury to the tendon of the long head of the biceps. In this prospective study, only patients who fulfilled the following criteria were included for analysis: (a) Patients presenting with an isolated type II SLAP lesion which was (b) verified by magnetic resonance arthrography (MRA), (c) who would be available for at least 24 months of follow-up after surgery and (d) for whom a complete data set was also available. Exclusion criteria were as follows: (a) The presence of any concomitant lesions, including a partial- or full-thickness rotator cuff tear, symptomatic acromioclavicular joint arthrosis, or a labral tear requiring a repair outside the SLAP region (i.e., the 10:30–1:30 “clock position” of the superior labrum), (b) additional repairs being performed at the time of surgery (e.g., rotator cuff repair, labrum repair outside the SLAP region, biceps tenodesis or tenotomy, or distal clavicle procedures), (c) a follow-up time of less than 24 months or being lost to follow-up, and (d) possession of an incomplete data set.

For the SLAP diagnosis 4 different evaluation criteria were used [18]. First, patient’s history with overhead activities, pain and clicking in abduction and external rotation as well as a sensation of instability were used as indicators for a potential SLAP lesion. Second, a clinical examination including ROM in all planes, impingement tests (Hawkins, Neer, Painful arc), instability tests (anterior apprehension and relocation), as well as biceps-related tests (active compression test, Yergason test, Jobe test full can) were carried out. As imaging method a MRA was performed which showed a separation of the labrum from the glenoid in the biceps anchor region. The radiographic determination of the SLAP lesion was made by a radiologist trained in musculoskeletal radiology. Diagnostic arthroscopy was used to confirm the separation of the labrum from the glenoid on the one hand by using a probe and on the other by provoking a peel back intraoperatively.

Preoperatively, 17 patients (20%) with an isolated type II SLAP lesion were included. Following arthroscopy, 5 patients were excluded due to either intraoperative findings (*n* = 3; 2 SLAP I lesions and 1 SLAP III lesion with a partial rotator-cuff tear) or other intraoperatively addressed pathologies (*n* = 2; 1 biceps tenotomy and 1 lateral clavicle resection). One patient was lost to follow-up, leaving 11 patients who were ultimately included in this study. All of these patients had a history of overhead activities (volleyball *n* = 5; climbing *n* = 2; tennis *n* = 2; hockey *n* = 1; yoga *n* = 1). Five patients (4 males, 1 female) performed their sports at competitive and 6 patients (4 males, 2 females) on recreational levels. The institutional review board (EK-No: 2010/355) approved this study and informed consent was obtained from all patients prior to the investigation.

### Methods

Functional assessments were performed preoperatively, and at 3, 6, 12 and >24 months postoperatively, using the following metrics: The Individual Relative Constant Score (CS_indiv_) [17], The American Shoulder and Elbow Surgeons Standardized Shoulder Assessment Form (ASES) [16], The Visual analogue scale (VAS) and The Short Form 36 (SF-36) [19].

For evaluation of the ability to return to pre-injury sports activities at final follow-up patients were preoperatively asked for their sports as well as their sports level, either competitive or recreational.

### Physical examination

All patients were examined by two independent, blinded observers (senior orthopedic and trauma surgeons) who were not involved in the primary treatment. The examination was based upon the aforementioned tests. Range of motion (ROM) measurements were performed using a handheld goniometer graded in single degrees, and assessment of isometric muscular strength was achieved using a dynamometer (Bonso Handy Scale, 15 kg capacity; 20 g graduation; ISO 9001; Bonso Electronics Ltd., Hong Kong, China) in abduction in the scapular plane – as suggested for the Constant Score [17].

### Surgical technique

**Fig. 1:**
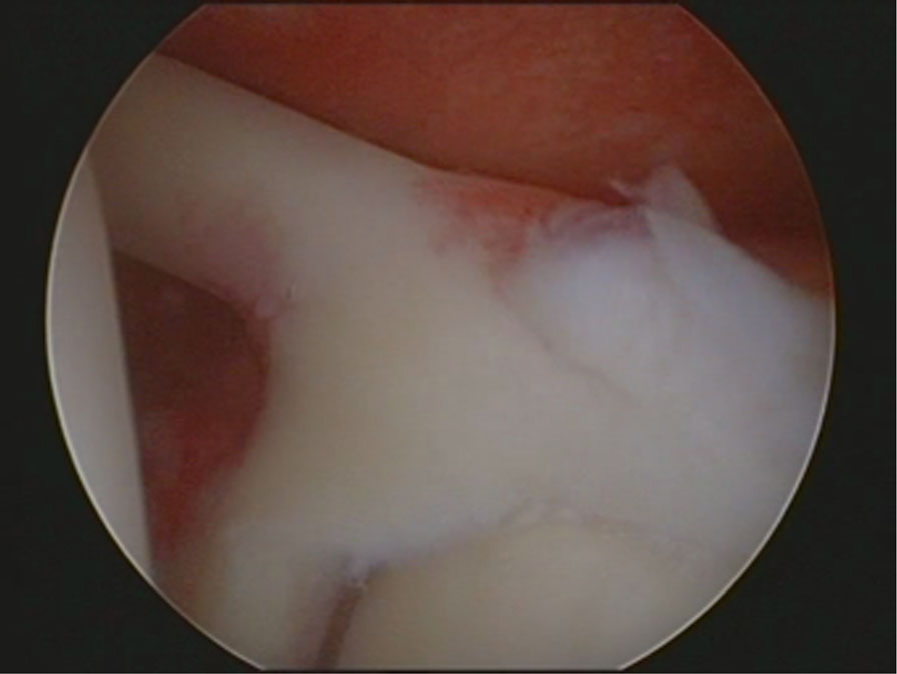
Diagnostic arthroscopy – Type II SLAP lesion

**Fig. 2:**
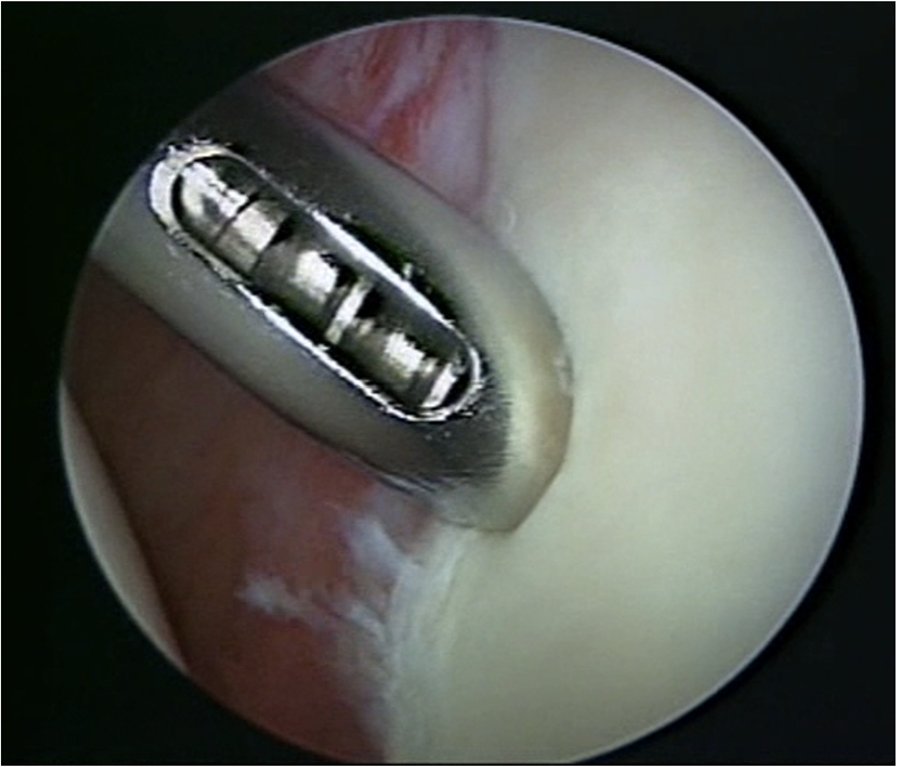
Inserting the metal screw anchor

**Fig. 3:**
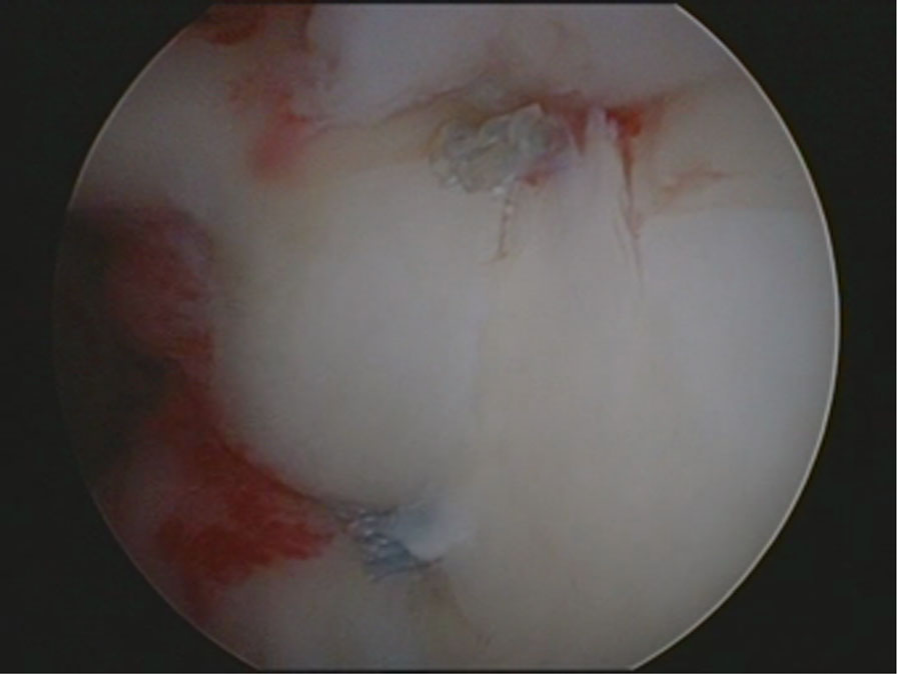
Restored labral complex

Two shoulder-fellowship-trained surgeons performed all arthroscopically assisted SLAP repairs. All surgeries were performed in beach chair position, starting with an arthroscopic diagnostic confirmation of the MRA findings (Fig. 1). If there was a gap between the glenoid and the labrum between 10 and 2 o’clock, tested by probe, the arm was brought in abduction and external rotation was performed to trigger the peel back sign. Thus proving the instability of the biceps tendon anchor, the superior labral complex was mobilized and debrided, if necessary. The superior glenoid rim was prepared with a rasp and then, using the Neviaser portal, a metal screw suture anchor (FASTak®, Arthrex Comp. Naples, FL, USA) single-loaded with a 2.0 suture (FiberWire®, Arthrex Comp. Naples, FL, USA) was inserted posterior to the biceps tendon anchor at 11 or 1 o’clock depending on the side of the shoulder (Fig. 2). The posterior labrum was secured with a mattress stitch and the arthroscopic knot was positioned superior to the tendon complex to avoid glenohumeral impingement (Fig. 3). The stability of the SLAP region was tested using a probe as well as repeating the peel back mechanism and if necessary, an additional anchor was placed anterior to the biceps tendon anchor at 1 or 11 o’clock in order to achieve a stable condition.

### Rehabilitation

All patients underwent a standardized postoperative rehabilitation protocol administered by this department. During the first two weeks, the shoulder was immobilized with an abduction sling, and patients were allowed to only remove their arm for hygiene and to perform daily pendulum exercises. Patients began physical therapy with 1 visit per week and started a home exercise program.

After removal of the abduction sling physical therapy was increased to 2 visits per week. Isometric exercises and passive ROM exercises for the elbow and hand, as well as hand-gripping exercises, were also allowed. Throughout week 3 and 4, ROM exercises were performed passively in the scapular plane, to approximately 45 degrees of internal rotation and to 60 degrees of elevation. Passive and active range of flexion was increased to 60, then 90, and ultimately >90 degrees, in 2 week intervals. Following the standard protocol at our institution, no external rotation or resisted biceps activity (both elbow flexion and forearm supination) was allowed for 8 weeks.

After regaining full ROM that is usually reached between week 8 and 12 under physical therapy, isometric strengthening and stretching exercises in all planes were started. Additionally, patients were advised to continue their home exercises including rotator cuff strengthening using therabands. According to the patients’ preference the strengthening training between month 4 and 6 after surgery was performed under physiotherapeutic guidance or by self-organized exercises. However, a return to sports typically occurred at approximately 6 months following surgery, after regaining pain-free function and strength.

### Statistics

The free software environment R version 3.0.2, as well as the lme4 package, were used for statistical analysis. Time courses were analyzed with linear mixed effects models, where random effects were defined by subject IDs and fixed effects by the second degree polynomial of approximate time in months. False discovery rate (FDR) corrected *p*-values of less than 0.05 were regarded as statistically significant.

In post-hoc analysis, the Wilcoxon signed-rank test for paired data was used for comparison of measurements at specific time points. Confidence intervals for the figures were estimated using 1000 bootstrap samples for each figure.

Since this study was designed as a case series, no formal power calculation was done a priori. Nevertheless, we empirically estimated power to detect a difference in scores between 6 months and a baseline visit, as well as between 3 months and a baseline visit, using 1000 bootstrap samples.

## Results

A total of 11 patients (92%) completed the study at a mean follow-up of 41.9 months (range: 36.1–48.4 months). There were 8 males and 3 females, with a mean age of 31.8 years (range: 22.8–49.8 years). About one third (*n* = 4; 36.4%) described a traumatic event to their shoulder as being the cause of the SLAP lesion. The remainder (*n* = 7; 63.6%) could not identify an isolated traumatic event as the cause of their shoulder injury. Patients’ main complaints at time of first presentation were pain and recurrent instability. 9 patients (81%) presented with an injury to the dominant extremity. In 7 patients 1 metal screw anchor was placed posterior to the biceps tendon anchor and in 4 patients 2 metal screw anchors, 1 anterior and 1 posterior to the biceps tendon anchor, were used for SLAP repair according to the size of the lesion (mean: 1.3 anchors). Patient demographics are presented in detail in Table 1.

**Table 1:** Patient demographics

Age, mean (range), years	31.8 (22.8 – 49.8)
Sex n (%)	
Male	8 (72.7)
Female	3 (27.3)
Follow-up, mean (range), months	41.9 (36.1 – 48.4)
Injury origin, n (%)	
Traumatic	4 (36.4)
Atraumatic	7 (63.6)
Injury to dominant extremity, n (%)	9 (81)
Number of anchors required for repair, mean (range)	1.33 (1-2)

**Fig. 4:**
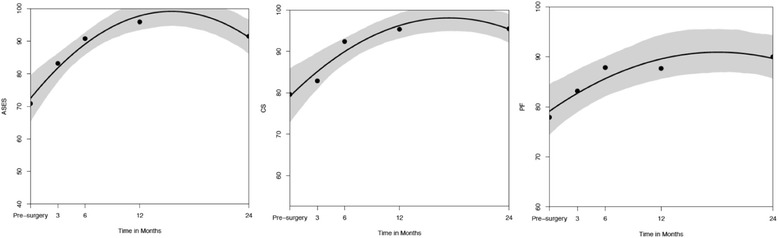
ASES, CS, and PF over time (pre-OP, 3, 6, 12, >24 months)

There was a statistically significant improvement of function according to the CS_indiv_ (*p* < 0.001), the ASES Score (*p* < 0.001), and the SF-36 “physical functioning” subscale (*p* < 0.001) (Fig. 4).

**Fig. 5:**
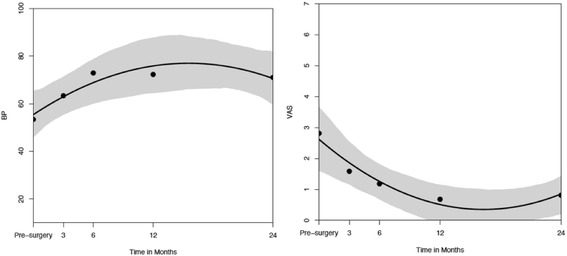
BP and VAS over time (pre-OP, 3, 6, 12, >24 months)

Additionally, there was a statistically significant decrease in pain according to the VAS (*p* < 0.001) and the SF-36 “bodily pain” subscale (*p* = 0.039) (Fig. 5). In the latter metric, score higher values indicate lower pain. Both pain parameters remained more or less stable after the 6-month check-up through the last follow-up although the pre-injury and for the injury responsible sport had been resumed.

The post-hoc analysis for comparison of pre- and postoperative functional outcome and pain scores showed a significant increase of function according to the CS_indiv_ (*p* = 0.004), the ASES Score (*p* = 0.006), and the SF-36 “physical functioning” subscale (*p* = 0.014) at 6 months postoperatively (Tables 2 and 4). Pain significantly decreased at 6 months postoperatively according to the VAS (*p* = 0.021), and at 12 months postoperatively in the SF-36 “bodily pain” subscale (*p* = 0.022) (Tables 3 and 4). All other parameters did not reach statistical significance. For these 5 parameters, the empirical powers to detect a significant difference between the follow-ups at 6 months and 3 months vs. baseline were 0.796 and 0.363, respectively.

**Table 2:** Presenting a detailed overview of CS and ASES values over time

Score	Mean (SD)	Median	Minimum	Maximum	*p*-value
CS					
Preop.	79.7 (14.43)	85.4	52.6	95.3	
3 months	83.39 (10.6)	85.4	60.6	96.0	0.160
6 months	91.88 (6.3)	93.1	82.2	100.0	0.004^a^
12 months	95.29 (4.8)	97.6	90.0	100.0	0.003^a^
>24 months	95.48 (5.88)	99.0	88.0	100.0	0.001^a^
ASES					
Preop.	71.1 (16.96)	75.0	40.0	93.0	
3 months	83.1 (11.34)	85.0	62.0	98.0	0.02
6 months	90.8 (9.43)	95.0	75.0	100.0	0.006^a^
12 months	96.2 (5.67)	100.0	83.0	100.0	0.002^a^
>24 months	91.5 (10.2)	95.0	71.6	100.0	0.003^a^

^a^significant compared to preoperative levels at a non-corrected significance level of 0.05

**Table 3:** Presenting a detailed overview of VAS values over time

Score	Mean (SD)	Median	Minimum	Maximum	*p*-value
VAS					
Preop.	2.82 (2.65)	3	0	7	
3 months	1.41 (1.56)	1	0	5	0.084
6 months	1.18 (1.45)	0.5	0	4	0.021^a^
12 months	0.68 (1.19)	0	0	3	0.007^a^
>24 months	0.82 (1.33)	0	0	4	0.010^a^

^a^significant compared to preoperative levels at a non-corrected significance level of 0.05

**Table 4:** Presenting a detailed overview of SF-36 values (PF; BP) over time

Score	Mean (SD)	Median	Minimum	Maximum	*p*-value
SF-36. Physical Functioning					
Preop.	78.0 (12.47)	80.0	60.0	100.0	
3 months	83.2 (11.13)	85.0	60.0	100.0	0.160
6 months	88.0 (9.75)	90.0	70.0	100.0	0.014^a^
12 months	87.7 (8.84)	85.0	70.0	100.0	0.007^a^
>24 months	90.0 (8.26)	90.0	75.0	100.0	0.004^a^
SF-36. Bodily Pain					
Preop.	53.5 (23.62)	52.0	20.0	100.0	
3 months	63.6 (25.67)	74.0	10.0	100.0	0.238
6 months	73.1 (24.38)	74.0	22.0	100.0	0.077
12 months	72.5 (18.52)	74.0	41.0	100.0	0.022^a^
>24 months	71.2 (21.37)	74.0	41.0	100.0	0.022^a^

^a^significant compared to preoperative levels at a non-corrected significance level of 0.05

Half of our patients played sports in a competitive capacity, and were able to return to their pre-injury sports levels (just as the other half of our patients were able to return to their recreational levels of activity) 6 months after surgery. The return to pre-injury sports level was determined by asking the patient for his sports activity and level at final follow-up. If the patient practiced the same sport at the same level (competitive or recreational) compared to the time before injury, a return to pre-injury sports level was assumed which has to be considered critically due to some recall bias [20].

There were no surgically related postoperative complications in this series. However, one patient presented with a thrombosis of the upper extremity shortly before his 6-month follow-up, after he had slept on the affected arm. The thrombosis was verified by ultrasound, and the patient immediately received an anti-thrombotic therapy. In the end, functional outcome and measurements were not influenced by this episode.

Two patients presented with pain at follow-up investigations. In one patient, persistent pain remained after surgery. As dictated by the symptoms, an MRA was performed to rule out a re-rupture. The SLAP repair appeared intact, as did the other structures. We therefore suspected the presence of a persisting acromio-humeral impingement, and offered the patient an arthroscopic treatment. The patient refused to undergo another arthroscopy and began intensive physical therapy. At the last follow-up, the patient presented with a reduction in pain and was satisfied with the overall result.

**Fig. 6:**
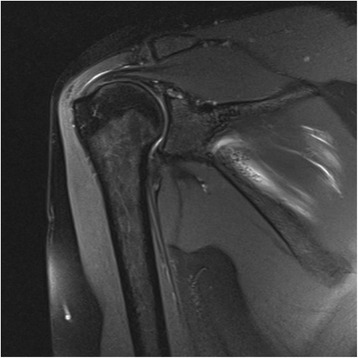
Magnetic resonance arthrography (MRA) of one patient 46 months after SLAP repair with the metal screw anchor in situ

One patient at final follow-up reported experiencing pain after taking part in intensive sports activity (volleyball). Due to the persistence of pain, an MRA was performed, which showed the healed type II SLAP lesion with the suture anchor in correct position – as well as a supraspinatus tendinopathy (Fig. 6). Apparently, the return to competitive training resulted in a physical overload with a consecutive lack of shoulder stabilization, thus resulting in an incipient impingement syndrome with a supraspinatus tendinopathy. The patient was advised to start physical therapy (back muscle training), which led to pain relief after the first cycle.

Both patients were able to return to their preoperative sports activities.

## Discussion

The most important finding of this study was that the arthroscopic repair of isolated type II SLAP lesions with metal screw suture anchors provides for satisfactory functional outcomes and a return to pre-injury sports levels at long-term follow-up. Although these rather satisfying results were not reached immediately after surgery, there was a significant increase of function and decrease in pain seen at 6 months postoperatively – a condition which thereafter remained stable, at least up through the last follow-up. To the best of our knowledge, this is the first study investigating the chronology of pain relief and return-of-function after anatomical SLAP repair using suture anchors. Our results indicate that a return to sports should not be allowed earlier than 6 months after surgery.

The largest study addressing the repair of isolated type II SLAP lesions was performed by Provencher et al. [8] who, using the BioSutureTak® (Arthrex, Naples, FL, USA), investigated 179 patients over a time period of 4 years. The authors identified an age of greater than 36 years to be a significant factor contributing to an increase of failure rate. In that series, a failure rate of 38% was observed, with 28% of patients requiring revision surgery. Schroder et al. [9] presented 102 patients treated by SLAP repair with resorbable tacks (Suretac; Acufex; Smith&Nephew) at a mean follow-up of 5 years. There were no significant differences between the results for patients 40 years of age or older and those under 40 years of age – which stands in contrast to the findings from Provencher et al. [8] In our series, we operated on a total of 3 patients over 40 years of age. All of these patients had a medical history of joint instability and recurrent pain and wanted to regain their pre-injury sports levels. When comparing patients over 40 years of age to those under 40, postoperatively we found no differences concerning functional outcome and pain relief.

Provencher et al. [8] reported that 13.1% of patients experienced difficulty with postoperative stiffness and pain. However, 88% of the patients were satisfied with their achieved results after a mean follow-up of 5 years [8]. In this series, we did not observe any postoperative stiffness. Revision surgery was not required for any patient through final follow-up.

Brockmeier et al. [12] reviewed 47 patients at an average follow-up of 2.7 years. The authors used bioabsorbable (*n* = 36) as well as metal anchors (*n* = 11) for SLAP repair, representing a significant increase of evaluated scores with respect to time. Although the ASES score was satisfying, with 93 points at final follow-up, only 74% of patients were capable of fully returning to their previous competitive level. The authors showed that patients presenting with a distinct traumatic aetiology had a greater likelihood of a successful return to sports [12]. In our study, patients reached a mean ASES score of 91.5 points, which is well in line with the results presented in the literature. Although all of our patients were able to return to their respective pre-injury sports level it has to be remarked that only few sports were represented and that our sample contained both competitive and recreational subjects with a wide range of ages. This 100% return to sports-rate would probably be different in a more homogeneous, younger group including pure overhead / throwing athletes [20].

Three authors prospectively reviewed patients older than 45 years [11, 13, 14] and did not find any advantages in repairing a type II SLAP lesion when associated with rotator cuff repair. Abbot et al. [11] demonstrated that in case of a minimally retracted rotator cuff tear combined with a SLAP II lesion rotator cuff repair and SLAP debridement leads to superior results regarding pain relief and function compared to rotator cuff and SLAP repair. Kanatli et al. [14] showed that arthroscopic repair of symptomatic type II SLAP lesions yields favourable outcomes in patients over 45 years of age, and the presence of accompanying rotator cuff tears had a negative effect on the results. Comparing rotator cuff repair combined with tenotomy versus SLAP repair Franceschi et al. [13] found significantly better clinical results after tenotomy in patients over 50 years of age.

In the present literature, the focus lies on clinical outcome measurement at a final follow-up of at least 2 years or greater [8–14]. All prospective studies [8–14] compare pre- and postoperative scores, as well as those at final follow-up. The pain component is only one of many elements built in different shoulder scores, although it is well known that patients complain of incessant pain after SLAP repair [21–24]. This fact represents a continuing challenge for the surgeon, the physical therapist and the patient himself.

Recommendations as to the right time point for a return to sports vary in the literature, ranging from 5 to 12 months after SLAP repair [8–10, 14, 25–28]. Others advocate full ROM, minimal pain, adequate strength and dynamic stability, and an appropriate rehabilitation progression [29] as indicators signalling the ability to return to preoperative sports activities. However, there is no general consensus about this.

In the present study, we took a closer look at the progression of function after isolated arthroscopic type II SLAP repair. We were able to show significant improvement according to the CS_indiv_, the ASES and the SF-36 “physical functioning” subscale at 6 months postoperatively – and remaining stable thereafter. We also separately investigated the progression of pain and found that in this sample pain decreased significantly at 6 months postoperatively according to the VAS and, at 12 months postoperatively according to the SF-36 “bodily pain” subscale. However, the VAS scores at 3 months were already trending towards significance and an increased sample size would probably show an earlier significant pain relief. Furthermore, it has to be considered, that this significant improvement from VAS 3 to VAS 1 out of 10 with average and median VAS scores of approximately 3 out of 10 represent mild to moderate levels of pain thus being uncomfortable but not clinically concerning.

Although biceps tenodesis or tenotomy [6, 7] lead to excellent functional results with an earlier relief from pain, SLAP repair remains the general recommendation in the young, active and demanding patient [30, 31]. Biceps tenodesis or tenotomy might serve as a secondary procedure if SLAP repair fails [32, 33], or as a primary procedure in patients over 60 years of age. In case of anatomical SLAP repair, the patient should be advised preoperatively that function and pain relief are likely to improve significantly at 6 months post-surgery – indicating the earliest point at which a return to pre-injury sports activities would be possible.

### Strengths and limitations

The major strengths are the prospective study design, a mean follow-up time of 3.5 years, and the assessment of patients’ outcomes with multiple validated outcome scores and physical examinations at fixed time points. The major limitations are the small study group limiting the generalizability of the results as well as the lack of a comparison group. Furthermore, our study group is very heterogeneous in means of performed sports activities both on competitive or recreational levels. The wide range of ages limits interpretability as the results may differ in a more homogeneous study group regarding sports activity, level, and age. Another point that has to be kept in mind is that pain itself is difficult to measure as there are many different factors influencing a person’s pain. Not only the extent of trauma or performed surgery exert an influence on the subjective sensation of pain but also central sensitization or psychological constructs.

## Conclusions

Arthroscopic repair of isolated type II SLAP lesions with suture anchors leads to a satisfactory functional outcome and a return to pre-injury sports levels with delayed but significant pain relief seen at 6 months after surgery. This indicates that a return to sports should not be allowed earlier than 6 months postoperatively, when the patient has regained pain-free function and recovered strength.

## Abbreviations

ASES: American Shoulder and Elbow Surgeons Standardized Shoulder Assessment Form
CS_indiv_: Individual Relative Constant Score
FDR: False discovery rate
MRA: Magnetic resonance arthrography
ROM: Range of motion
SF-36: The Short Form 36
SLAP: Superior labrum anterior to posterior
VAS: Visual analogue scale
